# Uncharted Waters: Treating Trauma Symptoms in the Context of Early Psychosis

**DOI:** 10.3390/jcm8091456

**Published:** 2019-09-12

**Authors:** Johanna B. Folk, Laura M. Tully, Dawn M. Blacker, Brandi D. Liles, Khalima A. Bolden, Valerie Tryon, Renata Botello, Tara A. Niendam

**Affiliations:** 1 Department of Psychiatry & Behavioral Sciences, University of California, Davis, School of Medicine, Sacramento, CA 95820, USA; johannafolk@gmail.com (J.B.F.); lmtully@ucdavis.edu (L.M.T.); kbolden@ucdavis.edu (K.A.B.); vltryon@ucdavis.edu (V.T.); rmbotello@ucdavis.edu (R.B.); 2 CAARE Center, Department of Pediatrics, University of California, Davis, Sacramento, CA 95820, USA; dmblacker@ucdavis.edu (D.M.B.); bliles@ucdavis.edu (B.D.L.)

**Keywords:** cognitive behavioral therapy, coordinated specialty care, early psychosis, empirically supported treatment, trauma

## Abstract

Psychosis is conceptualized in a neurodevelopmental vulnerability-stress framework, and childhood trauma is one environmental factor that can lead to psychotic symptoms and the development of psychotic disorders. Higher rates of trauma are associated with higher psychosis risk and greater symptom frequency and severity, resulting in increased hospitalization rates and demand on outpatient primary care and mental health services. Despite an estimated 70% of individuals in the early stages of psychosis reporting a history of experiencing traumatic events, trauma effects (post-traumatic anxiety or depressive symptoms) are often overlooked in psychosis treatment and current interventions typically do not target commonly comorbid post-traumatic stress symptoms. We presented a protocol for Trauma-Integrated Cognitive Behavioral Therapy for Psychosis (TI-CBTp), an approach to treating post-traumatic stress symptoms in the context of early psychosis care. We provided a brief summary of TI-CBTp as implemented in the context of Coordinated Specialty Care and presented preliminary data supporting the use of TI-CBTp in early psychosis care. The preliminary results suggest that individuals with comorbid psychosis and post-traumatic stress symptoms can be appropriately and safely treated using TI-CBTp within Coordinated Specialty Care.

## 1. Introduction

Psychosis is conceptualized in a neurodevelopmental vulnerability-stress framework whereby “vulnerability” comprises neurobiological abnormalities originating from genetic factors, abnormalities in fetal brain development and neuromaturational processes during adolescence and “stress” comprises environmental factors increasing risk for, or triggering the manifestation of, psychosis [1]. Early exposure to traumatic events is one environmental factor that can lead to psychosis [2,3]. Risk increases in a dose-related fashion; greater childhood trauma exposure is associated with higher psychosis risk [4] and symptom frequency and severity [5,6]. Research regarding the relationship between experiencing trauma during adulthood and psychotic symptoms is limited and mixed, though generally supports a positive association between adult traumatic experiences and severity of psychosis symptoms [7,8]. This results in increased demand across mental health services [9]. Comorbidity is also high; approximately 70% of individuals with early psychosis (EP) have a trauma history [10]. Although several Empirically Supported Treatments (ESTs) for trauma-related disorders exist, few are integrated into EP care [11].

To address this critical gap, we developed an integrated protocol for treating post-traumatic stress and psychosis symptoms in EP. Supported by Health Resources & Services Administration funding, the protocol was developed as part of the Trauma Adolescent Mental Illness predoctoral psychology internship at the University of California, Davis (UCD)—a collaboration between the Child and Adolescent Abuse Resource and Evaluation Diagnostic and Treatment (CAARE) Center and the Sacramento Early Diagnosis and Preventative Treatment (SacEDAPT) Clinic. EP includes recent onset of a psychotic disorder in the past two years (ROP) and clinical high risk for psychosis (CHR) based on subthreshold psychosis symptoms and/or genetic risk plus functional decline [12]. Building upon ESTs, our Trauma-Integrated Cognitive Behavioral Therapy for Psychosis (TI-CBTp) protocol integrates the coordinated specialty care (CSC) model for EP [13], cognitive-behavioral therapy for psychosis (CBTp) [14], and trauma-focused CBT treatments, including Trauma-Focused CBT (TF-CBT) [15], Prolonged Exposure Therapy (PE) [16], and Cognitive-Processing Therapy (CPT) [17]. The goal of the current manuscript was to provide a brief summary of TI-CBTp and to present preliminary data supporting the feasibility and impact of using TI-CBTp in EP care. Symptom and psychosocial functioning data were collected as part of standard clinical care at baseline (intake to clinic) and at 6- and 12-month follow-up assessments. A detailed description of the TI-CBTp approach and an illustrative example are presented in the Supplementary Material.

## 2. The Current Approach: TI-CBTp

TI-CBTp combines multiple ESTs (CBTp; TF-CBT, PE, CPT; CSC) to meet the needs of EP populations. CBTp, an EST for first episode psychosis [14] and youth at clinical high-risk [18,19,20], is used to address psychotic and common comorbid mood and anxiety symptoms. Psychotic symptoms are conceptualized as distressing and stigmatizing interpretations of intrusions on normal awareness [21]; cognitive and behavioral techniques are used to evaluate the content and accuracy of these interpretations and generate alternative interpretations to reduce distress and improve functioning. Core CBTp elements include psychoeducation, case conceptualization, cognitive and behavioral interventions, and relapse management [14]. ESTs for post-traumatic stress symptoms are also used and involve four key elements: psychoeducation, skill building, exposure, and safety planning [14]. Psychoeducation normalizes responses to traumatic events and describes common reactions to trauma, types of traumatic events, and post-traumatic stress disorder (PTSD). Skill building teaches patients to replace maladaptive coping skills with more adaptive skills to manage post-traumatic stress symptoms. Exposure facilitates desensitization to trauma-related cues, reduces associated negative emotions (e.g., shame, helplessness) and allows for processing of trauma-related memories. Safety enhancement/relapse management solidifies skills such as abuse prevention and healthy relationships.

Within the context of this paper, we described implementation of TI-CBTp within the UCD SacEDAPT Clinic, which is an outpatient setting, using the CSC model. We decided to implement TI-CBTp within the CSC model because it addresses clinical and functional impacts of psychosis via a multidisciplinary treatment team to comprehensively support the client and the family’s needs. Key components include rapid identification after psychosis onset, comprehensive assessment, psychiatric and medical management, supported employment and education, family support, psychoeducation, CBT, substance use management, and suicide prevention [13]. Family members or other support persons are key participants in CSC and family-related interventions include psychoeducation, communication, problem solving, and relapse prevention. It may also be appropriate to use TI-CBTp in other settings where longer-term treatment relationships can be established, although this remains an empirical question.

**Figure 1:**
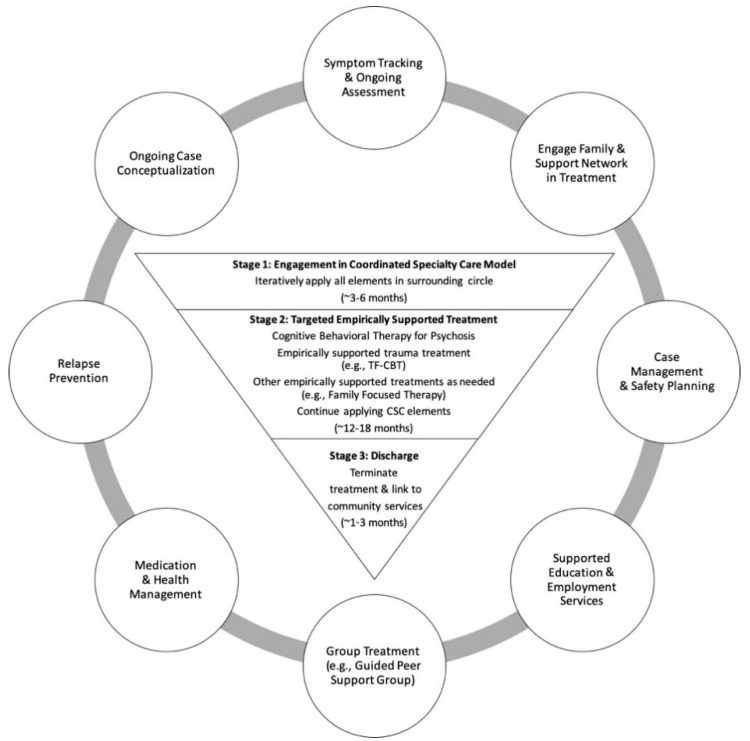
Integration of Cognitive-Behavioral Therapy for Psychosis and Empirically Supported Trauma Treatment within Coordinated Specialty Care: A Three-Stage Model. *Note*. CSC = Coordinated Specialty Care; This figure represents the three-stage approach to integrating Cognitive Behavioral Therapy for Psychosis and empirically supported trauma treatments within CSC. Key components of CSC are depicted in the outer circle as they should continue throughout the three stages. The three stages build upon one another, with each serving as a necessary foundation for subsequent stages.

Figure 1 illustrates how CBTp and ESTs for post-traumatic stress symptoms can be integrated into the typical course of treatment in a CSC program. We outlined the treatment approach in three stages. Stage 1 involves initial engagement of the client and family in all elements of the CSC model, general psychoeducation about psychosis, and ongoing assessment of symptoms to prompt an initial case conceptualization to guide treatment, coupled with risk management and safety planning as needed. These Stage 1 activities can last approximately 3–6 months, depending on the client’s needs, and are conducted in parallel and iteratively (represented as a continuous cycle in Figure 1). Once clients are stabilized and engaged in treatment, they transition to Stage 2, where conceptualization driven CBTp, ESTs for post-traumatic stress symptoms, and other ESTs as needed, can be implemented. Stage 2 can last approximately 12–18 months and should be integrated with ongoing activities described in Stage 1. Stage 3 reflects the period of time (approximately 3 months) just prior to a client graduating from a CSC program, when clients, families, and treatment teams engage in discharge planning and linking to community care.

**Figure 2:**
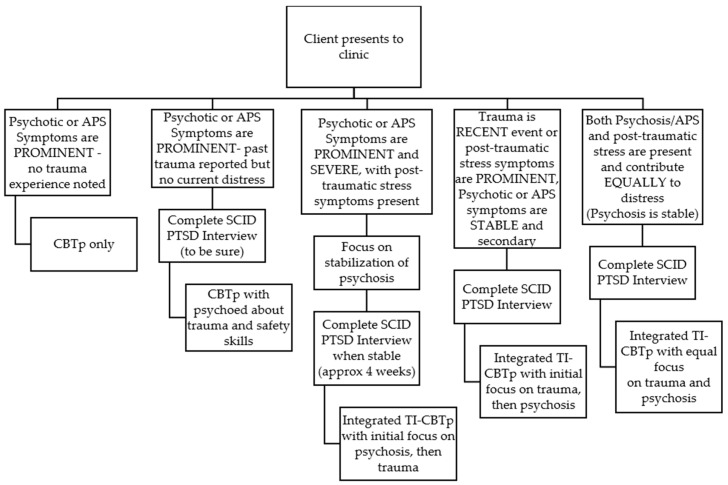
Decision tree to guide clinical approach. *Note.* APS = Attenuated positive symptoms; CPTp = Cognitive-behavioral therapy for psychosis; SCID = Structured Clinical Interview for DSM Disorders; PTSD = Post-traumatic stress disorder; TI-CBTp = Trauma-integrated cognitive-behavioral therapy for psychosis.

The TI-CBTp protocol outlines three steps prior to beginning targeted empirically supported psychosis and trauma treatment (Stage 2). The first is determining whether psychosis or post-traumatic stress symptoms are the primary concern or equally distressing. Figure 2 provides a decision tree to guide the treatment approach. The second is identifying and addressing factors that could impede (e.g., suicidal ideation/behavior, repeated crises) or enhance (e.g., family support) treatment progress. The third is achieving a period of relative stability (i.e., one month on a stable medication dose, psychotic symptoms managed without significant distress or effect on behavior, and no engagement in high risk behavior) is requisite to proceeding with the trauma components of TI-CBTp. These steps were all followed in the current evaluation of TI-CBTp.

As illustrated in Figure 1, TI-CBTp implementation within the UCD SacEDAPT Clinic at Stage 2 of treatment included the following key components: psychoeducation, skill building, exposure, and relapse management and enhancing safety (see Supplemental Material for detailed descriptions). Within the TI-CBTp framework, *psychoeducation* is used to normalize responses to traumatic events or psychosis onset and reinforce accurate cognitions about what occurred; it is emphasized at the start of treatment and integrated throughout. *Skill building* involves managing concerning symptoms and reactions to discussing traumatic experiences by replacing maladaptive with more adaptive relaxation, affect regulation, and cognitive coping skills relevant to various situations. *Exposure* unpairs thoughts, reminders, and memories from overwhelming negative emotions and breaks patterns of avoidance. TI-CBTp uses gradual exposure and in vivo exposure (as needed). Clients create a hierarchy and trauma narration (see TF-CBT manual [15]), to process “hot spot” memories. After narration, cognitive processing is used to explore and correct trauma-related cognitive errors. When appropriate, a support person is present as the client reviews their narration. Conjoint work facilitates communication about traumatic events. *Relapse management and enhancing safety* is the final stage and is used to reinforce relapse management and safety skills, create a transition plan for ongoing care, and ensure that the client has sufficient social supporters who are aware of and incorporated into a safety plan.

## 3. Experimental Section

### 3.1. Participants and Study Design

Individuals were enrolled in treatment at the UC Davis SacEDAPT Clinic from July 2014 to August 2018 and data was collected as part of standard clinic intake practices. Eligible individuals were between 12 and 30 years old and diagnosed with both psychotic and post-traumatic stress symptoms. Individuals completed assessments at baseline (intake to clinic) and 6- and 12-months post-baseline with masters and doctoral level clinicians as part of ongoing clinical care. Primary assessments were the SCID [22,23], Structured Interview for Prodromal Symptoms [12], Clinical Global Impressions Scale [18,24], Global Functioning Social and Role (GFS, GFR) Scales [25], the Global Assessment of Functioning [26], the Insight item from the Positive and Negative Syndrome Scale (PANSS; higher scores reflect greater insight impairment) [27], and the Columbia Suicide Severity Rating Scale [28]. Procedures for assessing post-traumatic stress symptom severity changed during the course of data collection, resulting in the use of multiple measures. Adults completed the PTSD Checklist for DSM-5 [29] and youth completed the UCLA PTSD Reaction Index [30] or the Child and Adolescent Trauma Screen [31]. Data was collected during standard clinical practice as part of quality improvement and was approved by the UC Davis Institutional Review Board. De-identified data was provided for this preliminary analysis.

### 3.2. Analysis

Baseline sample characteristics were examined using descriptive statistics and differences between the two groups (Stage 1, Stage 2) were tested using independent samples t-tests for continuous variables (e.g., age), Chi-Square tests for nominal data (e.g., sex), and Mann–Whitney U tests for ordinal data (e.g., Clinical Global Impressions Scale scores). Ordinal logistic regressions were conducted for each ordinal outcome variable (e.g., Clinical Global Impressions Scale scores, insight) to examine changes over time and differences between Stage 1 and Stage 2 clients; a two-way, repeated measures ANOVA was conducted for the continuous Global Assessment of Functioning outcome. Due to missing data for the majority of clients, changes in post-traumatic symptom severity are presented for descriptive purposes. Since three different trauma symptom measures were used depending on when the client entered the clinic and their age, scores were normalized to the maximum score for each respective measure.

## 4. Results

### 4.1. Sample Characteristics

Between July 2014 and August 2018, 22 individuals (19 ROP; 3 CHR) were identified as having co-morbid psychosis and post-traumatic stress symptoms at baseline (intake to UCD SacEDAPT Clinic). All these individuals started within Stage 1 of TI-CBTp (Figure 1). Twelve clients remained in Stage 1 at the time of analysis and 10 individuals moved on to Stage 2 of the protocol. All clients participated in elements of CSC (Stage 1), regardless of participation in targeted empirically supported psychosis and trauma treatment (Stage 2). Table 1 displays the baseline demographic characteristics for Stage 1 and Stage 2 clients; there were no statistically significant differences in baseline characteristics between the two groups.

### 4.2. Outcomes

Ordinal logistic regressions were used to assess differences in changes in symptoms and functioning over time between Stage 1 and Stage 2 clients. Time point (baseline, 6 months, and 12 months) and stage (Stage 1 or 2) were the predictors in the model for each outcome variable (Clinical Global Impressions Scale, Global Functioning Social and Role Scales, Insight).

**Figure 3:**
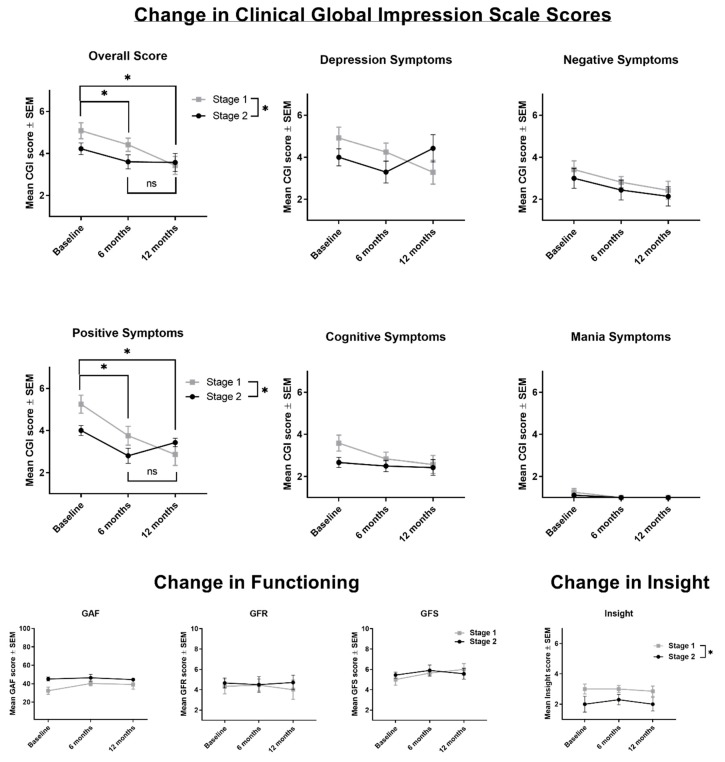
Changes in outcomes across 12 months of treatment. *Note.* GAF = Global Assessment of Functioning; GFR = Global Functioning Role Scale; GFS = Global Functioning Social Scale; NSSIB = Non-suicidal self-injurious behavior. Stage 1 *n* = 12; Stage 2 *n* = 10. *N*s differed by time point. For the overall, positive, depression, mania, and cognitive CGI data, the *n*s were as follows: Overall: Stage 1: 12 (baseline), 12 (6 months), 7 (12 months); Stage 2: 9 (baseline), 10 (6 months), 7 (12 months). For negative CGI scores: Stage 1: 12 (baseline), 11 (6 months), 7 (12 months); Stage 2: 9 (baseline), 9 (6 months), 7 (12 months).). For the functioning data, the *n*s were as follows: GAF: Stage 1: 12 (baseline), 12 (6 months), 7 (12 months); Stage 2: 10 (baseline), 10 (6 months), 7 (12 months). GFR: Stage 1: 12 (baseline), 11 (6 months), 7 (12 months); Stage 2: 9 (baseline), 10 (6 months), 7 (12 months). GFS: Stage 1: 12 (baseline), 11 (6 months), 6 (12 months); Stage 2: 9 (baseline), 10 (6 months), 7 (12 months). For Insight, *n*s were: Stage 1: 12 (baseline), 11 (6 months), 7 (12 months); Stage 2: 6 (baseline), 10 (6 months), 7 (12 months).

Three main findings emerged (Figure 3). First, the ordinal regression model significantly predicted overall symptoms on the Clinical Global Impressions Scale, χ^2^ (3) = 14.49, *p* = 0.002; pseudo *R*^2^ = 0.24. The odds ratio of having higher positive symptoms on the Clinical Global Impressions Scale for Stage 1 versus Stage 2 was 2.98 (95% CI, 1.10 to 8.02), a statistically significant effect, Wald χ^2^ (1) = 4.65, *p* = 0.03. Overall, symptom severity significantly differed according to the time points at which symptoms were measured, Wald χ^2^ (2) = 9.16, *p* = 0.002, with baseline symptoms being significantly higher than 6 months (*p* = 0.04) and 12 months (*p* = 0.002). The odds ratio of having more severe overall symptoms on the Clinical Global Impressions Scale at baseline compared to 6 months was 3.37 (95% CI, 1.07 to 10.62).The odds ratio of having more severe overall symptoms on the Clinical Global Impressions Scale at baseline compared to 12 months was 7.87 (95% CI, 2.07 to 29.94). Six month and 12 month symptom severity did not significantly differ (*p* = 0.19).

Second, the ordinal regression model significantly predicted positive symptoms on the Clinical Global Impressions Scale, χ2 (3) = 21.33, *p* = 0.000; pseudo R2 = 0.33. The odds ratio of having higher positive symptoms on the Clinical Global Impressions Scale for Stage 1 versus Stage 2 was 3.54 (95% CI, 1.31 to 9.60), a statistically significant effect, χ^2^ (1) = 6.17, *p* = 0.01. Positive symptom severity significantly differed by time point at which symptoms were measured, Wald χ^2^ (2) = 11.07, *p* = 0.001, with baseline symptoms being significantly higher than 6 months (*p* = 0.001) and 12 months (*p* = 0.001). The odds ratio of having more severe positive symptoms at baseline compared to 6 months was 7.91 (95% CI, 2.33 to 26.82). The odds ratio of having more severe positive symptoms at baseline compared to 12 months was 10.31 (95% CI, 2.61 to 40.78); 6-month and 12-month symptom severity did not significantly differ (*p* = 0.67).

Finally, the ordinal regression model significantly predicted insight symptoms, χ^2^ (3) = 8.42, *p* = 0.04; pseudo *R*^2^ = 0.16. The odds ratio of having less insight for Stage 1 versus Stage 2 was 4.34 (95% CI, 1.51 to 12.50), a statistically significant effect, Wald χ^2^ (1) = 7.42, *p* = 0.006. Insight scores did not change over time (*p* = 0.65).

Stage and time point were not significant predictors for any other outcome measures (GF:S, GF:R, CGI negative, depression, mania, or cognitive scores, all ps >0.10). A two-way repeated measures ANOVA was run to assess the difference in GAF score over time between Stage 1 and Stage 2 clients. There was no effect of time (F (2, 18) = 0.52, *p* = 0.60) or stage (F (1, 18) = 2.67, *p* = 0.12) on GAF scores, and no significant interaction (*p* = 0.29).

**Figure 4:**
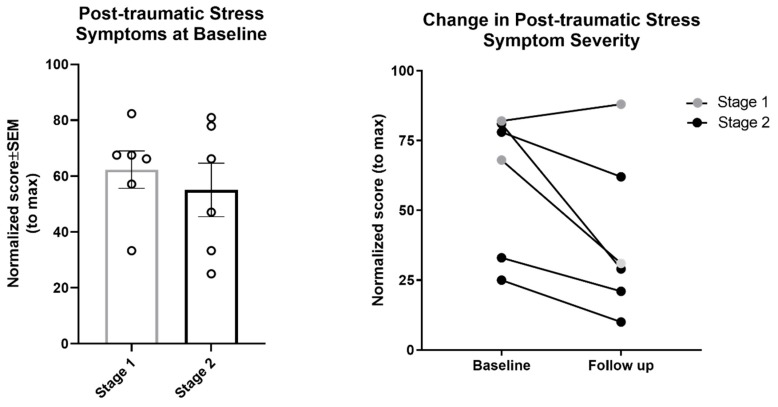
Post-traumatic stress symptom severity at baseline (Stage 1: *n* = 6; Stage 2: *n* = 6) and 6-month follow-up (Stage 1: *n* = 2; Stage 2: *n* = 4). Post-traumatic stress symptoms were measured on the UCLA PTSD Reaction Index or the Child and Adolescent Trauma Screen (for minors), or the PTSD Checklist for DSM-5 (for adults). Scores were normalized to the maximum score for each respective measure.

As shown in Figure 4, post-traumatic stress symptom severity tended to decline from baseline to follow-up among Stage 2 clients (one Stage 1 client showed a slight increase). Due to the small sample sizes, these data are provided for descriptive purposes only; no statistical tests were conducted.

## 5. Discussion

These preliminary results suggest that individuals with psychosis and post-traumatic stress symptoms can be appropriately and safely treated using TI-CBTp within a CSC model; the data indicate no detrimental effects or exacerbation of psychotic symptoms—a common concern raised when discussing trauma treatment in psychosis populations [11]. Of the 22 individuals who received TI-CBTp, 45% (*n* = 10) progressed from Stage 1 of CSC care to Stage 2 to receive targeted empirically supported trauma and psychosis treatment. Preliminary data suggest that those who proceed to Stage 2 of TI-CBTp tend to have lower positive symptoms, higher overall functioning, and better insight at baseline; all CHR clients in this sample progressed to Stage 2, compared with 37% of the ROP clients. These characteristics may allow them to capitalize more quickly on the skills learned and services provided in Stage 1 and enable movement into trauma-integrated treatment. In contrast, individuals who are coping with more severe symptoms and poorer functioning may need a longer period of support in Stage 1 to stabilize clinically before shifting the core focus of treatment to post-traumatic stress symptoms. Other factors, such as family support, housing stability, and access to resources and basic needs (food security, transport, etc.), may have also contributed to the successful transition from Stage 1 to 2, but data on these social determinants were not available for this analysis.

Clients who moved to Stage 2 demonstrated a reduction in clinical symptoms over the course of one year of treatment, with little change in functioning or insight. Those who remained in Stage 1 also demonstrated improvements in clinical symptoms, consistent with evidence supporting the CSC model as an effective intervention for EP [11]. Although the lack of a control group precludes our ability to draw conclusions regarding whether Stage 2 interventions reduce symptoms to a greater extent than Stage 1 interventions, the preliminary data suggest that treatment of comorbid trauma symptoms in the context of psychosis does not lead to clinical worsening. In contrast, our findings suggest that TI-CBTp may serve as an appropriate method of addressing comorbid psychosis and post-traumatic stress symptoms in the context of EP care.

### 5.1. Limitations and Future Directions

The results of the current study are preliminary and intended to inform future large-scale trials of TI-CBTp. Limitations reflect the realities of data collection as part of real-world community-based clinical care in the absence of research funding. Key limitations include the lack of a random assignment or no-treatment control group, a small sample size from a single clinic, the inability to account for treatment dosage (e.g., number of sessions), and limited data on changes in post-traumatic stress symptoms. Although post-traumatic stress symptoms were assessed for all clients at baseline, clinic procedures changed during the data collection period resulting in the use of multiple measures [29,30,31], and follow-up data are limited. Consequently, we cannot draw any conclusions regarding the effect of TI-CBTp on comorbid trauma symptoms. Strengths include the implementation of TI-CBTp in a real-world community-based clinical setting, the use of empirically validated assessments and evaluation of a novel treatment approach to meet the needs of EP populations. Future randomized clinical trials are recommended to address the aforementioned limitations and expand our knowledge regarding the effectiveness of TI-CBTp.

### 5.2. Challenges of Treating Trauma in Early Psychosis

TI-CBTp requires flexible application of CBTp and trauma treatment within a complex multi-need population. Within the context of this pilot trial, we encountered many challenges in implementing TI-CBTp in a community-based EP program. We highlighted some of these challenges below as the field considers how best to meet the needs of individuals with comorbid trauma and psychosis symptoms.

#### 5.1.1. Cognitive Symptoms

Executive functioning impairment is common in psychosis [32] and negatively impacts sustained attention/memory, ability to follow instructions, and problem solving. We recommend that clinicians provide manageable amounts of information per session using concrete examples, visual aids, and repetition. For clients who have difficulty remembering to complete assignments, it may be helpful to problem-solve ways in which the support person can help. Cognitive remediation [33] may be needed as an adjunct service to improve cognitive functioning.

#### 5.1.2. Negative Symptoms

Negative symptoms impair quality of life, interfere with treatment, and are not addressed by anti-psychotic medication. Negative symptoms can be addressed through behavioral activation, mastery and pleasure ratings for activities, and emotion induction. For individuals with poverty of speech, clinicians may need to use alternative mediums for narration, such as drawing or writing.

#### 5.1.3. Recurrent Crises

Unmet psychosocial needs and risk concerns can derail treatment course because clinicians can focus on responding to ongoing crises [15]. Recurrent crises may reflect avoidance of trauma-treatment, or instability in the client’s and/or family’s life. We found that addressing these concerns before trauma-integrated treatment, and especially prior to gradual exposure, can minimize the likelihood of treatment derailment. Case management may be needed to support clients and family members with psychosocial, financial or unmet mental health needs. ESTs should be used flexibly to address underlying problems behind repeated crises. Achieving at least one month of relative stability before exposure may decrease the likelihood that these concerns will arise and impact treatment, although this remains an empirical question.

#### 5.1.4. Reality of Incident

If a clinician is unsure whether a trauma has occurred or if it was a psychotic experience, collateral information and records should be collected, when possible, as these can provide an outside perspective and/or objective evidence regarding an incident. If the event did not occur, trauma processing can focus on the distress around the traumatic delusion or hallucination rather than the event itself. When clients achieve clinical stability, they may exhibit insight into whether the event was real or a psychotic experience. We advise clinicians to proceed carefully and not make assumptions about the validity of the report, as this runs the risk of invalidating the client’s experience.

#### 5.1.5. Clinician Skepticism

Clinicians are skeptical about the appropriateness of exposure-based interventions for individuals with EP and comorbid traumas symptoms [11]. Four studies evaluating the effect of exposure in adults with PTSD and chronic psychotic disorders demonstrated that CBT, PE, and eye movement desensitization and reprocessing were safe and effective at reducing PTSD and psychotic symptoms [14,34,35,36]. Although no published studies evaluated the effectiveness of exposure-based therapy for trauma-related disorders in EP, experts agree that trauma-focused treatment should be used as a first-line treatment for clients at all stages of psychosis [11].

#### 5.1.6. Secondary Traumatic Stress

Clinicians often experience secondary traumatic stress from hearing about clients’ traumatic experiences. According to the National Child Traumatic Stress Network [37], 6%–26% of clinicians working with traumatized populations are at high-risk for secondary traumatic stress, compromising professional functioning and diminishing quality of life. Preventative strategies should include regular self-report assessments of secondary traumatic stress, participation in workplace self-care groups, caseload balancing, flextime scheduling, and use of the self-care accountability system.

#### 5.1.7. Training Recommendations

TI-CBTp integrates elements of multiple treatment protocols, and as such, the authors recommend that clinicians receive extensive training in CBTp and at least one trauma treatment protocol (TF-CBT, PE, or CPT) prior to implementing TI-CBTp. Training in DBT principles would also be beneficial, although it is not required. Consultation calls with experts in the above models are often part of the training experience and can help clinicians generalize components to specific cases.

## 6. Conclusions

There are currently no evidence-based treatments for comorbid trauma-related disorders in the early stages of psychosis. Individuals with psychosis are routinely excluded from trauma research and practice, severely limiting our understanding of appropriate treatment. Our preliminary work using TI-CBTp suggests that trauma-integrated services for clients with EP is feasible. Efforts to systematically/empirically evaluate TI-CBTp on a larger scale are underway to inform future training of clinicians in providing trauma-informed services for clients with psychosis.

## Figures and Tables

**Table 1:** Baseline demographic characteristics.

		Stage 1 *N* = 12	Stage 2 *N* = 10
Age at assessment *M* (*SD*)	Years	19.1 (5.4)	16.8 (4.9)
Sex *N* (%)	Female	7 (58%)	7 (70%)
	Male	5 (42%)	3 (30%)
Race *N* (%)	Black	6(50%)	4 (40%)
	White	2 (17%)	2 (20%)
	Other Asian	0 (0%)	1 (10%)
	Other Pacific Islander	1 (8%)	0 (0%)
	Other Race	3 (25%)	3 (30%)
Ethnicity *N* (%)	Other Hispanic/Latino	4 (33%)	3 (30%)
	Not Hispanic	8 (67%)	7 (70%)
Primary Language *N* (%)	English	12 (100%)	10 (100%)
Diagnosis *N* (%)	Mood with Psychotic Features	5 (42%)	3 (30%)
	Schizophrenia Spectrum Disorders	7 (58%)	3 (30%)
	Other Specified/Not Otherwise Specified Schizophrenia Spectrum	0 (0%)	4 (40%)
PTSD Diagnosis *N* (%)	PTSD Dx	7 (58%)	6 (60%)
Post-traumatic Stress Symptom Severity *M (SD)*	Normalized Score	62.3 (16.4)	55.1 (23.5)
Psychosis Diagnosis *N* (%)	Recent Onset	12 (100%)	7 (70%)
	Clinical High Risk	0 (0%)	3 (30%)
Substance Use *N* (%)	Lifetime	6 (50%)	3 (30%)
	Current	5 (42%)	2 (20%)
Suicidal Ideation *M* (*SD*)	Lifetime	3.3 (2.0)	2.2 (2.4)
	Current	1.0 (1.3)	0.4 (1.0)
Suicidal Behavior *N* (%)	Lifetime	5 (42%)	4 (40%)
	Current	0 (0%)	1 (10%)
Non-Suicidal Self-Injury *N* (%)	Lifetime	5 (42%)	4 (40%)
	Current	1 (8%)	2 (20%)
Insight *M* (*SD*)	Current	3.0 (1.1)	2.0 (1.3)

On the Clinical Global Impressions Scale, clients in both stages demonstrated clinically significant overall symptoms at baseline (Stage 1: *M* = 5.1; *SD* = 1.3; range = 2–7; Stage 2: *M* = 4.2; *SD* = 0.8; range = 3-5). Clients in the Stage 1 group demonstrated statistically higher levels of positive (U = 22.0, *p* = 0.019) and overall (U = 28.5, *p*= 0.049) symptoms compared to the Stage 2 group; there were no group differences in negative, depressive, mania, or cognitive symptoms. Clients also exhibited serious psychosocial impairments on the Global Assessment of Functioning (Stage 1 *M* = 32.3; *SD* = 13.3; Stage 2 *M* = 40.3, *SD* = 9.0), Global Functioning Role Scale (Stage 1 *M* = 4.3; *SD* = 2.6; Stage 2 *M* = 4.7, *SD* = 1.5), and Global Functioning Social Scale (Stage 1 *M* = 5.0; *SD* = 1.9; Stage 2 *M* = 5.4, *SD* = 0.9). Clients in the Stage 1 group had statistically significant lower Global Assessment of Functioning scores (*t* = −2.85, *p* = 0.011) than Stage 2 clients; social and role functioning did not significantly differ between the groups.

## Supplementary Materials

The following are available online at https://www.mdpi.com/2077-0383/8/9/1456/s1, Supplementary Materials: Detailed Description of Treatment.
