# Supplementary material for: Uncharted Waters: Treating Trauma Symptoms in the Context of Early Psychosis
Source: J Clin Med. 2019 Sep 12;8(9):1456. doi: 10.3390/jcm8091456 (PMC6780072; doi:10.3390/jcm8091456)
Supplement: Supplementary file 1 [file jcm-08-01456-s001.pdf]

Supplement: Detailed Description of Treatment

Trauma-Integrated Cognitive Behavioral Therapy for Psychosis (TI-CBTp) integrates multiple Empirically Supported Treatments (Trauma Focused Cognitive Behavior Therapy [1], Prolonged Exposure [2], Cognitive Processing Therapy [3]) to meet the needs of Early Psychosis (EP) populations. TI-CBTp emphasizes broad skill building (relaxation, affect regulation, cognitive coping) to support EP individuals in managing psychosocial stressors and symptoms; a combination of imaginal and in-vivo gradual exposure, depending on the client's needs, is used to practice skills and achieve desensitization, while managing any recurrence of psychotic symptoms to avoid relapse. TI-CBTp focuses heavily on safety enhancement and relapse management, as EP individuals are at high-risk for maltreatment throughout their lives, and facilitates linkage to long-term care as needed. Given the importance of family support in EP outcomes [4-5], TI-CBTp involves support persons on some level throughout treatment regardless of the client's age. Below we outline the key components of TI-CBTp: assessment, formulation and treatment planning, psychoeducation, skill building, exposure, and relapse management and enhancing safety.

1. Assessment

Comprehensive psychodiagnostic assessment facilitates understanding symptoms (e.g., duration, severity, onset, frequency, triggers) and functional impairment. Individuals with psychosis can experience cognitive impairments impacting memory and attention [6], so collateral information can be critical to initial diagnosis and case conceptualization.

Psychosis. The Structured Clinical Interview for DSM-5 (SCID-5) [7] can be used to comprehensively assess psychotic and comorbid symptoms. For attenuated psychotic symptoms, the Structured Interview for Psychosis-Risk Symptoms [8] is recommended. The Clinical Global Impression Scale [9] or Colorado Symptom Index Scale [10] can be used to monitor symptoms over time.

Trauma Events and Effects. For adults, the SCID-5 or the PTSD Checklist for DSM-5 (PCL-5) [11] can be used to assess post-traumatic stress symptoms. For children and adolescents, the UCLA PTSD Reaction Index for DSM-5 [12] or the Child and Adolescent Trauma Screen [13] assess exposure to traumatic events and PTSD criteria. To monitor progress, post-traumatic stress symptoms should be assessed at intake, before exposure, and after completing trauma-focused treatment. Meeting full criteria for PTSD is not a pre-requisite for proceeding with TI-CBTp, as individuals with post-traumatic stress symptoms that do not meet the diagnostic threshold can still potentially benefit from trauma-focused treatment. Furthermore, the timing of a traumatic experience (childhood versus adulthood), though relevant to case conceptualization, is not a determining factor in whether TI-CBTp is appropriate; this approach can be used whether the client experienced trauma during childhood, adulthood, or both.

Risk. Trauma and psychosis are associated with increased suicide risk [14–18]; standardized risk assessment during the intake and throughout treatment is critical. The Columbia Suicide Severity Rating Scale [19] quantifies the severity of suicidal ideation and behavior over the lifetime and in the past month. Alternatives include the Collaborative Assessment and Management of Suicidality [20] and the Linehan Risk Assessment and Management Protocol [21], which include guidance for managing suicide risk.

Culture and Language. The DSM-5 Cultural Formulation Interview [22] can be used to understand the individual's cultural background and perspective on their symptoms and experiences. Within the context of psychosis, an individual's cultural perspective may influence whether an experience is abnormal or distressing. For example, if the experience of communicating with ancestors is a common cultural experience and does not cause distress or impairment, it would not be conceptualized as a psychotic symptom. Similarly, language can impact the expression of an individual's experience and contribute to misinterpretations of experience as more or less significant. Therefore, completing

assessments in an individual's primary language (using an interpreter if needed) can help to avoid miscommunication. One's cultural beliefs may also impact whether an experience is seen as traumatic. The experience of war by a large group of individuals may lessen their perception of the experience as a trauma. Similarly, the use of corporal punishment is not seen as physical abuse by some cultural groups. Therefore, understanding the individual's cultural view of the traumatic event can impact its conceptualization and the treatment approach.

2. Formulation and Treatment Planning

Clinicians should formulate an initial case conceptualization following the assessment and determine whether psychosis or post-traumatic stress symptoms are the primary concern or equally distressing (Figure 2 in main text). It is important to note that case conceptualization is an iterative process; clinicians should revisit their conceptualization throughout treatment as additional information about the client is obtained. Additional factors that could impede or enhance treatment progress should also be identified early-on in treatment. Example impeding factors include unmet basic needs, severe substance dependence, low motivation and engagement in treatment, repeated crises, and significant family-child problems including potential loss of placement. We recommend addressing problems in this order:

1. Suicidal ideation/behavior, danger to others, nonsuicidal self-injury
2. Therapy interfering behavior (e.g., repeated no shows, repeated crises)
3. Central problem (e.g., trauma, psychosis, both)
4. Substance use (Could fall into any above. If not, address last)

3. Psychoeducation

Psychoeducation normalizes responses to traumatic events or psychosis onset and reinforces accurate cognitions about what occurred. Psychoeducation is emphasized at the start of treatment and integrated throughout. We provide some specific guidance regarding psychoeducation and encourage clinicians to consult CSC [23], CBTp [24], and trauma-related protocols [1-3] for further details and examples.

Assessment Feedback and Treatment Planning helps clients and families understand relevant symptom dimensions and risk behaviors, identify treatment goals and the most distressing symptoms and/or traumatic events, and determine level of involvement by family members/support persons.

Crisis/Relapse Planning requires clients and families to understand symptoms and behaviors interfering with clinical stability (e.g., harm to self/others, substance use, psychosis). Safety assessment must occur throughout treatment. Case management needs must be addressed early to avoid impact on treatment engagement; establishing relative clinical stability allows client engagement. "Relative stability" is defined here as at least one month on a stable medication dose, psychotic symptoms managed without significant distress or effect on behavior, and no engagement in high risk behavior. Once relative stability is achieved, relapse planning should occur so clients can monitor symptoms and respond appropriately.

General Psychoeducation teaches clients and families about symptoms and provides gradual exposure through educational material in order to normalize and validate the client's experiences. Normalization of the event and associated effects include helping the client feel they are not alone in the experience and the associated symptoms are common. Validation includes helping the client understand that their thoughts, feelings, and behaviors make sense in the context of their experiences and may have been used in order to survive. The goal of psychoeducation is not for clients to process the details of their

own traumatic events or to create a narrative of the client's life or traumatic experiences. This is because 1) clients may not have the skills to process all the memories at this time, which may lead to dissociation or overwhelming emotions; and 2) clients may not have a strong therapeutic relationship with the clinician at this early point in treatment. If clients spontaneously disclose trauma-related information during psychoeducation, it is important to normalize emotional responses to psychoeducation, validate the client's distress, and move on. Clinicians might also need to set boundaries if clients bring up many details about prior traumas; this should be done sensitively and through reminding the client why you are not engaging in processing of their specific traumatic experience at this stage.

Psychosis facts and symptoms. Clients and families should understand how and why symptoms develop and worsen, including positive, negative, and cognitive symptoms of psychosis, depression and anxiety, and the vulnerability-stress model [23, 25]. To track triggers, patterns, and treatment progress, clients should monitor symptoms and behaviors via a diary card [41], worksheet (e.g., a simple table with columns to record the triggering event, resulting emotion, and behavioral response), or smartphone application, and discuss each session. For example in Dialectical Behavior Therapy [26], diary cards are used to track suicidal ideations and actions, self-harm urges and actions, other risk behaviors, and emotion intensity experienced throughout the week. Trigger monitoring includes the situation in which the trigger occurred, thoughts about what caused the trigger, emotions elicited during the trigger, and positive and negative consequences of behaviors during or after the trigger. Inclusion of support persons is recommended, particularly when cognitive symptoms impede ability to complete assignments.

Trauma and post-traumatic stress symptoms. Trauma psychoeducation should include information about the event(s), how many people experience similar traumatic events (e.g. prevalence of childhood sexual abuse), common perpetrators and their behaviors, thoughts/feelings/reactions after trauma, how trauma effects the brain, and how treatment will facilitate symptom reduction [1]. Clients have often experienced multiple traumatic events and didactic instruction on each one may be impractical. The focus of psychoeducation and exposure should be events that are most distressing and impairing to the client. The National Child Traumatic Stress Network [27] is one common resource which provides worksheets and handouts that can be used during psychoeducation about trauma events and effects.

4. Skill Building

TI-CBTp skill building includes managing concerning symptoms and reactions to discussing traumatic experiences by replacing maladaptive with more adaptive coping skills relevant to various situations.

Relaxation Skills. People who have experienced trauma often have chronic heightened physiological reactions to stress, responding as if a traumatic event is reoccurring. Clients should learn skills for both non-crisis and crisis situations. Non-crisis relaxation skills include breathing, progressive muscle relaxation, and mindfulness. Crisis relaxation skills can include Dialectical Behavior Therapy [26] distress tolerance skills (e.g., TIPP, self-soothe, IMPROVE, Radical Acceptance) to increase tolerance of painful emotions without engaging in potentially harmful behavior.

Affect Regulation. People who have experienced trauma and/or psychosis typically have a predominance of painful emotions and affect dysregulation or may experience significant negative symptoms (e.g., anhedonia, avolition). Affect regulation skills help with emotion identification, affective expression, managing negative emotions, problem solving, social skills, and positive self-talk. Behavioral activation can increase energy and drive when negative symptoms are present; emotion induction can facilitate connections to emotional experiences.

Cognitive Coping. Cognitive coping allows exploration of thoughts and correction of inaccurate or unhelpful cognitions. Psychosis- or trauma-related cognitions should not be challenged at this time; this can be invalidating and ineffective until after gradual exposure. Cognitive coping increases awareness of

one's internal dialogue, beginning with non-trauma or non-psychosis related examples. Clients learn the connection between thoughts, feelings and behaviors using the cognitive triangle. Clients also learn "catch it, check it, change it" to identify negative automatic thoughts (catch), examine their accuracy and helpfulness (check), and restructure unhelpful/inaccurate thoughts (change).

5. Exposure

Exposure unpairs thoughts, reminders, and memories from overwhelming negative emotions and breaks patterns of avoidance. Within a CBT framework, avoidance of trauma-related stimuli maintains post-traumatic stress symptoms and worsens distress over time. Exposing individuals to trauma reminders imaginally or in vivo in a safe environment reduces fear and avoidance [28].

TI-CBTp uses gradual exposure. Clients create a hierarchy and trauma narrative (see TF-CBT manual [1). For complex trauma, creating a timeline of events can help identify the most salient traumatic experiences, and themes (e.g., trust, safety) are used in narration along with specific events. Clients process "hot spot" memories, not their entire lifetime, for 6 to 8 sessions. After narration, cognitive processing is used to explore and correct trauma-related cognitive errors.

When appropriate, the support person is present as the client reviews their narration. Conjoint work facilitates communication about traumatic events. We recommend clinicians share details of the traumatic events with support persons at least 2-3 times prior to the conjoint session; this allows time for support persons to process their own reactions and prepare supportive or validating statements. In some cases a support person may not be appropriate for this process for various reasons and the narrative can be shared with the clinician only. For adults who do not wish to share their narration, a broader discussion of the trauma with a support person is recommended to develop a shared understanding of the traumatic event and how to support the client's coping skills.

TI-CBTp also uses in vivo exposure to reduce post-traumatic stress and psychotic symptoms. Exposures can be conducted during or between sessions. For example, to test the belief "If I go outside, my voices will get worse," a client might walk around the block and record how frequently they hear voices; this data can be used to challenge their belief and generate an alternative belief. Clients' post-traumatic stress and psychosis symptom reduction should be assessed after exposure to evaluate effectiveness of treatment.

6. Relapse Management and Enhancing Safety

This final stage reinforces relapse management and safety skills. Clients and families should be well-versed in identifying signs of worsening symptoms or risk behaviors and comfortable using coping skills. When relevant, clients should have reduced substance use or self-harm behavior and increased healthy behaviors. Relapse management solidifies these skills and helps clients and families realize symptoms may worsen and they have appropriate ways of coping or seeking care. Common safety and self-care skills are reinforced at this stage (e.g., abuse prevention, healthy relationships, bullying, safe sex). These skills are useful for everyone, but essential to EP clients who are at high-risk for future maltreatment and re-hospitalization. Conjoint sessions can be helpful for discussing the family safety plan, managing future stress, and identifying family and community supports.

A transition plan for ongoing care is advisable; mandatory if the client is taking medication. New providers should understand what symptoms are better attributed to trauma vs. psychosis (e.g., hypervigilance vs. paranoia). A written transition summary of what TI-CBTp treatment entailed is helpful. Whenever possible, a joint session between current and new provider should occur to maintain engagement.

Supplement: Case Illustration of TI-CBTp Implementation

Background

Jabar (pseudonym) is a 25 year-old Syrian male (fluent in English) referred to the UCD SacEDAPT clinic following an inpatient hospitalization. Jabar completed a comprehensive intake assessment including the SCID-5 and PCL-5. Information from the initial referral suggested Jabar was experiencing full-threshold psychotic symptoms. Based on Jabar's report at intake, he began experiencing attenuated positive symptoms in the form of somatic distortions approximately two and half years earlier. Six months later, he began hearing voices (auditory hallucinations) and believing people were out to get him (paranoid delusions). Jabar became increasingly paranoid over time, believing a microchip was implanted in his brain to monitor him. Jabar's paranoid ideation became so distressing he quit his job to avoid people at work who might have been targeting him. He became homeless and began seeing what he described as ghosts (visual hallucinations). His symptoms persisted over a two-year period and he was hospitalized four times due to danger to self. Jabar reported long-standing depressive symptoms including depressed mood, diminished interest, increased appetite with weight gain, psychomotor retardation, loss of energy, feelings of worthlessness, difficulty concentrating, and suicidal ideation and behavior. He experienced four major depressive episodes. Regarding trauma exposure history, Jabar was born in a war zone and forcibly displaced during childhood. He grew up with a physically abusive mother who abused alcohol. Jabar temporarily lived in a refugee camp and was adopted by a couple in the United States along with his three siblings during his early adolescence. Jabar reported intense psychological distress and physiological arousal when exposed to reminders of the traumatic events (e.g., being in the presence of an intoxicated person), persistent and exaggerated negative beliefs about others and the world, persistent fear, an exaggerated startle response, hypervigilance, and irritability. He reported no current or previous substance abuse or dependence. On the Columbia Suicide Severity Rating Scale [36], Jabar endorsed nonspecific active suicidal thoughts in the past month, and in his lifetime, one suicide attempt and two periods of preparatory behavior. Jabar's sister and her boyfriend, with whom he resided, provided collateral perspectives. Jabar's family was supportive, although significant family stress and high levels of emotional expression often resulted in family conflict. Based on the symptoms described above, Jabar was diagnosed with schizoaffective disorder, depressive type, and PTSD.

Treatment Plan

Jabar's psychotic symptoms were prominent and severe, with post-traumatic stress symptoms also present. Prior to beginning trauma-focused treatment, the clinical team determined it was necessary to stabilize his psychosis. Jabar's chronic suicidal ideation and the high levels of expressed emotion within his family were also of concern as these could hinder treatment progress. It was determined additional family therapy would be needed to support Jabar's clinical stability and treatment progress. As part of the CSC model, Jabar participated in weekly individual therapy, weekly peer group, bi-weekly Multi-Family Group [40], monthly psychiatry services, and case management as needed to support his vocational, financial, and recreational goals.

Psychoeducation

Jabar's initial sessions focused on reviewing intake assessment results, psychoeducation around his diagnoses, and the treatment approach provided by the SacEDAPT Clinic. Suicidal ideation was also addressed early in treatment through creation of an individualized suicidal ideation rating scale (like a subjective units of distress scale) and a safety plan. Jabar identified coping skills to use at different levels

of suicidal ideation severity. His family received instruction on effective ways to communicate about suicide, and his environment was modified to increase safety (e.g., locking up sharp objects, restricting driving). Guidance for when to seek emergency intervention was provided. Jabar's family was highly involved in treatment, gaining skills to support his clinical stability and treatment progress.

Jabar was initially adherent to the prescribed atypical antipsychotic medication, reporting reduced psychotic and depressive symptoms, and less intense and less frequent suicidal ideation. During this period of relative stability, he proceeded with general psychoeducation about psychosis and trauma. Over time he became less willing to endure the medication's side effects and stopped taking them, which led his somatic hallucinations to recur. The team provided psychoeducation about medication side effects and the process of working with the treatment team to find the right medication, and supported Jabar in advocating for himself to try new medications. After several medication trials, his symptoms stabilized on an antipsychotic and antidepressant with side effect profiles he considered tolerable.

General psychoeducation began with a focus on symptoms of psychosis. Jabar identified his negative symptoms as the most impairing/distressing as they made getting out of bed and engaging in goal-related activity challenging. His family viewed this as "laziness," and his impaired short-term memory as "avoidance;" psychoeducation was provided to the family to address these assumptions. Jabar's suspiciousness lead to anxiety in social situations, where he frequently worried he was being judged or discriminated against. Jabar's suspiciousness was partially grounded in his past traumatic experiences and the ongoing climate of racism in his community; distinguishing between psychotic, trauma-related, and reality-based suspiciousness was not simple. Jabar was taught to identify symptoms of anxiety and to more objectively evaluate safety within his environment. He received psychoeducation about exposure and was encouraged to participate in a weekly peer group at the clinic to increase his exposure to social situations.

Jabar identified his experiences with physical abuse and parental substance abuse as most distressing. Psychoeducation included learning about PTSD, the neurobiology of trauma (e.g., fight/flight/freeze response) and activities including the "What do you know?" game where he learned about the prevalence of physical abuse and why it occurs. Jabar's clinician consulted with an expert on working with refugees. This expert cautioned that although Jabar's warzone experiences may sound traumatic to someone who was not raised in that environment, for him this may have been the norm and as such, not distressing or impairing in the way one might imagine. She advised some psychoeducation about warzone trauma be covered, but with less of a focus given Jabar did not endorse significant resultant distress.

Skill Building

At this point, Jabar had developed coping skills to manage his suicidal ideation. For example, he regularly practiced meditation to clear his head of negative thoughts and distressing emotions. Additional relaxation skills including controlled breathing and progressive muscle relaxation were reviewed. During affect regulation, behavioral activation was crucial for Jabar as he continued to struggle with anhedonia and avolition. Jabar began regularly exercising, and increasing his engagement in activities to build mastery (e.g., caring for chickens at his house) and pleasure (e.g., spending time outside with his dog). Jabar was familiar with the cognitive model from prior treatment and began completing thought records and identifying the accuracy and helpfulness of his thoughts. One challenge for Jabar was remembering to complete thought records between sessions, as his cognitive symptoms impacted his memory. He engaged in problem-solving across multiple sessions regarding strategies for improving his memory and enlisted the help of his family in providing reminders.

Exposure

Jabar identified four traumatic incidents/themes for his narration: physical abuse intertwined with his mother's alcohol abuse, forced migration, trust, and safety. Jabar did not exhibit avoidance in beginning the narration, but struggled to remember details of specific events due to cognitive symptoms. This made identifying "hot spots" challenging; Jabar's sister provided the clinician with additional details about their childhood experiences to facilitate the process.

Jabar struggled to emotionally connect with the material due to affective blunting caused by his medication. In collaboration with Jabar's prescribing psychiatrist, emotional induction (through watching video clips and identifying body cues experienced) was used during narration to help Jabar connect with feelings of sadness and anger. The Assessment of Post-Narrative Emotional State [44] was used to help Jabar identify his experiences with a wide range of emotions, rating them on a 1 to 10 scale.

During the post-exposure reassessment, Jabar reported he was not experiencing any clinically significant trauma or psychosis symptoms. His functioning had also improved; he was preparing to go back to work and increasing the frequency and quality of his social interactions.

Relapse Management and Enhancing Safety

A wellness plan [45] was completed with Jabar to help him recognize early warning signs of relapse, situations/events that may trigger relapse, ways to cope in triggering situations and when early warning signs appear, and a safety plan in case of full relapse. He identified skills/activities he uses (e.g., medication adherence, exercise) to stay well. Much of this information was covered throughout treatment; this process served to reinforce this material.

Bolstering family support was key for Jabar. Through participation in multi-family group and collateral sessions, Jabar's sister and her boyfriend learned more effective communication skills to discuss stressful and traumatic subjects. The family planned to discuss how everyone is coping with ongoing stressors on a weekly basis, as well as more specifically about Jabar's and his sister's management of trauma reminders.

References:

1. Cohen, J. A.; Mannarino, A. P.; Deblinger, E. *Treating Trauma and Traumatic Grief in Children and Adolescents.*; Guilford Press: New York, NY, 2017.

2. Foa, E. B.; Hembree, E. A.; Rothbaum, B. . *Prolonged Exposure Therapy for PTSD*; Oxford University: New York, 2007.

3. Resick, P. A.; Schnicke, M. *Cognitive Processing Therapy for Rape Victims: A Treatment Manual*; Sage Publications: Newbury Park, CA, 1993.

4. McFarlane, W. R.; Link, B.; Dushay, R.; Marchal, J.; Crilly, J. Psychoeducational Multiple Family Groups: Four-Year Relapse Outcome in Schizophrenia. *Fam. Process* **1995**, *34*, 127–144. https://doi.org/10.1111/j.1545-5300.1995.00127.x.

5. O'Brien, M. P.; Zinberg, J. L.; Bearden, C. E.; Daley, M.; Niendam, T. A.; Kopelowicz, A.; Cannon, T. D. Psychoeducational Multi-Family Group Treatment with Adolescents at High Risk for Developing Psychosis. *Early Interv. Psychiatry* **2007**, *1*, 325–332. https://doi.org/10.1111/j.1751-7893.2007.00046.x.

6. Heinrichs, R. W.; Zakzanis, K. K. Neurocognitive Deficit in Schizophrenia : A Quantitative Review of the Evidence. *Neuropsychology* **1998**, *12* (3), 426–445. https://doi.org/http://dx.doi.org/10.1016/j.knosys.2006.03.007.

7. First, M. B.; Williams, J. B. W.; Karg, R. S.; Spitzer, R. L. *Structured Clinical Interview for DSM-5—Research Version*; American Psychiatric Association: Arlington, VA, 2015.

8. McGlashan, T. H.; Walsh, B. C.; Woods, S. W. *Structured Interview for Psychosis-Risk Syndromes*. PRIME Research Clinic: New Haven, CT, 2014.

9. Spearing, M. K.; Post, R. M.; Leverich, G. S.; Brandt, D.; Nolen, W. Modification of the Clinical Global Impressions (CGI) Scale for use in bipolar illness (BP): the CGI-BP. *Psychiatry Res* **1997,** *73*, 159-171.

10. Shern, D. L.; Wilson, N. Z.; Coen, A. S.; Patrick, D. C.; Foster, M.; Bartsch, D. A.; Demmler, J. Client Outcomes II: Longitudinal Client Data from the Colorado Treatment Outcome Study. *Milbank Q.* **1994**, *72* (1), 123–148. https://doi.org/10.2307/3350341.

11. Weathers, F. W.; Litz, B. T.; Keane, T. M.; Palmieri, P. A.; Marx, B. P.; Schnurr, P. P. The ptsd checklist for dsm-5 (pcl-5) Scale available from the National Center for PTSD at www. ptsd. va. gov.

12. Steinberg, A. M.; Brymer, M. J.; Kim, S.; Briggs, E. C.; Ippen, C. G.; Ostrowski, S. A.; Gully, K. J.; Pynoos, R. S. Psychometric Properties of the UCLA PTSD Reaction Index: Part I. *J. Trauma. Stress* **2013**, *26*, 1–9. https://doi.org/10.1002/jts.21780.

13. Sachser, C.; Berliner, L.; Holt, T.; Jensen, T. K.; Jungbluth, N.; Risch, E.; Rosner, R.; Goldbeck, L. International Development and Psychometric Properties of the Child and Adolescent Trauma Screen (CATS). *J. Affect. Disord.* **2017**, *210*, 189–195. https://doi.org/10.1016/j.jad.2016.12.040.

14. Castelein, S.; Liemburg, E. J.; De Lange, J. S.; Van Es, F. D.; Visser, E.; Aleman, A.; Bruggeman, R.; Knegtering, H. Suicide in Recent Onset Psychosis Revisited: Significant Reduction of Suicide Rate over the Last Two Decades - A Replication Study of a Dutch Incidence Cohort. *PLoS One* **2015**, *10* (6), 1–13. https://doi.org/10.1371/journal.pone.0129263.

15. Conus, P.; Cotton, S.; Schimmelmann, B. G.; Berk, M.; Daglas, R.; McGorry, P. D.; Lambert, M. Pretreatment and Outcome Correlates of Past Sexual and Physical Trauma in 118 Bipolar I Disorder Patients with a First Episode of Psychotic Mania. *Bipolar Disord.* **2010**, *12*, 244–252. https://doi.org/10.1111/j.1399-5618.2010.00813.x.

16. Dube, S. R.; Anda, R. F.; Felitti, V. J.; Chapman, D. P.; Williamson, D. F.; Giles, W. H. Childhood Abuse , Household Dysfunction , and the Risk of Attempted Suicide Findings From the Adverse Childhood Experiences Study. *JAMA* **2001**, *286* (24), 3089–3096.

17. Ösby, U.; Correia, N.; Brandt, L.; Ekbom, A.; Sparén, P. Mortality and Causes of Death in Schizophrenia in Stockholm County, Sweden. *Schizophr. Res.* **2000**, *45*, 21–28. https://doi.org/10.1016/S0920-9964(99)00191-7.

18. Zuschlag, Z. D.; Korte, J. E.; Hamner, M. Predictors of Lifetime Suicide Attempts in Individuals With Attenuated Psychosis Syndrome. *J. Psychiatr. Pract.* **2018**, *24* (3), 169–178.

19. Posner, K.; Brown, G. K.; Stanley, B.; Brent, D. A.; Yershova, K. V; Oquendo, M. A.; Currier, G. W.; Glenn Melvin, M. A.; Greenhill, L.; Shen, S.; et al. The Columbia–Suicide Severity Rating Scale: Initial Validity and Internal Consistency Findings from Three Multisite Studies with Adolescents and Adults. *Am. J. Psychiatry* **2011**, *168*, 1266–1277.

20. Jobes, D. A.; Comtois, K. A.; Brenner, L. A.; Gutierrez, P. M.; O'Connor, S. S. Trials of the Collaborative Assessment and Management of Suicidality (CAMS). In *The International Handbook of Suicide Prevention*; Rory O'Conner, Jane Pirkis, Eds.; Wiley Blackwell: Hoboken, NJ, 2016; pp 431–449.

21. Linehan, M. M.; Comtois, K. A.; Ward-Ciesielski, E. F. Assessing and Managing Risk With Suicidal Individuals. *Cogn. Behav. Pract.* **2012**, *19*, 218–232. https://doi.org/10.1016/j.cbpra.2010.11.008.

22. American Psychiatric Association. *Diagnostic and Statistical Manual of Mental Disorders*; American Psychiatric Publishing: Arlington, VA, 2013.

23. Heinssen, R. K.; Goldstein, A. B.; Azrin, S. T. *Evidence-Based Treatments for First Episode Psychosis: Components of Coordinated Specialty Care*; Bethesda, MD, 2014.

24. Hutton, P.; Taylor, P. J. Cognitive Behavioural Therapy for Psychosis Prevention: A Systematic Review and Meta-Analysis. *Psychol. Med.* **2014**, *44*, 449–468. https://doi.org/10.1017/S0033291713000354.

25. Mueser, K. T.; Deavers, F.; Penn, D. L.; Cassisi, J. E. Psychosocial Treatments for Schizophrenia. *Annu. Rev. Clinincal Psychochology* **2013**, *9*, 465–497. https://doi.org/10.1177/0963721410377743.

26. Linehan, M. M. *DBT Skills Training Manual*, 2nd ed.; The Guilford Press: New York, NY, 2015.

27. The National Child Traumatic Stress Network. Available online: https://www.nctsn.org (accessed 15 July 2019).

28. Foa, E. B.; Keane, T. J.; Friedman, M. J. *Effective Treatments for PTSD: Practice Guildelines from the International Society for Traumatic Stress Studies*; Guilford Press: New York, NY, 2000.
